# Improvement in Patient Satisfaction and Anxiety With Perioperative Music Therapy in Patients Undergoing Total Abdominal Hysterectomy: A Single-Blind Prospective Study

**DOI:** 10.7759/cureus.39519

**Published:** 2023-05-26

**Authors:** Aparna Shukla, Nishant Kaushik, Hemlata Hemlata, Reetu Verma, Shefali Gautam, Gyan Prakash Singh

**Affiliations:** 1 Department of Anaesthesiology, King George's Medical University (KGMU), Lucknow, IND

**Keywords:** spinal anaesthesia, visual analogue scales, total abdominal hysterectomy, music, therapy

## Abstract

Introduction

Listening to music is a safe and low-cost way to reduce preoperative anxiety among patients, but more research is needed to evaluate its effectiveness fully.

Aims

The aim of the study is to identify the effect of intraoperative music therapy on the visual analogue scale for anxiety (VASA) scores (VASA 1 and VASA 2) and patient satisfaction score (PSS) perioperatively.

Methods

In a study of 188 patients aged 40-70, those in group A (94 patients) listened to pre-approved music during their surgery for abdominal hysterectomy, while group B (94 patients) did not. Both groups wore noise-cancelling earphones. VASA was recorded before (VASA 1) and after (VASA 2) the surgery. PSS was recorded in the postoperative ward. Music preferences were kept confidential from the investigator recording the scores.

Result

The two groups of patients had similar demographic profiles and baseline characteristics.

The VASA 1 of both groups was similar, with a mean value of 4.36 ± 1.13 for group A and 4.23 ± 1.05 for group B (p = 0.606). However, group A had lower VASA 2 (1.79 ± 0.83) than group B (3.77 ± 0.98). The difference was statistically significant (p < 0.001).

The patient satisfaction score in group A was notably higher than those in group B. A total of 52 patients were highly satisfied in group A as compared to none in group B (p < 0.001), and a total of 42 patients were moderately satisfied as compared to eight patients in group B (p < 0.001). Eighty-six patients in group B were unsatisfied.

Conclusion

According to our research, playing specific music at the right volume can significantly lower anxiety levels and increase patients' satisfaction scores for those who have had abdominal hysterectomy surgeries.

## Introduction

The most routine gynaecological surgery performed worldwide is a total abdominal hysterectomy. However, adverse emotional reactions are usually associated with this surgery, which influences postoperative healing physically and emotionally [1].

Various drugs such as benzodiazepines have been tried to attenuate stress and anxiety intraoperatively. The reported incidence of adverse effects is variable as different dosages and combinations of drugs are used. Few studies showed decreased intraoperative anxiety with music therapy in patients undergoing various surgeries [2-4].

We aimed to evaluate the effects of music on the visual analogue scale for anxiety (VASA) scores (VASA 1 and VASA 2) intraoperatively and postoperatively and to assess the patient satisfaction score (PSS) postoperatively in patients undergoing total abdominal hysterectomy under spinal anaesthesia [5,6].

## Materials and methods

This prospective, randomised, single-blind study was conducted in a tertiary care centre in India for 18 months. After approval from the Institutional Ethical Committee of King George's Medical University (1778/Ethics/2021) and Clinical Trials Registry-India (CTRI/2022/07/043883), 188 American Society of Anesthesiologists (ASA) grade I and II patients between the age group of 40 and 70 years scheduled for total abdominal hysterectomy under spinal anaesthesia were included in the study. The exclusion criteria included contraindications to neuraxial anaesthesia, patient with hearing abnormality, patient with chronic pain syndrome and patient with mental or psychiatric disorders. After obtaining written consent from the patients, they were randomly assigned into two groups, with 94 patients in each group, using the sealed envelope technique for randomisation [7]. Group A patients received pre-approved music intraoperatively after a successful subarachnoid block (SAB). In contrast, group B patients did not have any music playing. However, all patients were equipped with earphones set to noise-cancellation mode for the duration of their surgery. Prior to their surgery, the patients were requested to provide a list of their preferred songs. These songs were downloaded and approved by the patients, and the volume was adjusted to their comfort level in the evening itself. Music preferences were kept confidential from the investigator documenting the scores. The patients were also explained about VASA score and PSS in the evening.

The VASA tool features a 10 cm line horizontally, with '0' on the far left to indicate no anxiety and '10' on the far right to represent the worst possible anxiety. The patient satisfaction score (PSS) was evaluated on a scale of 1-3, with '1' reflecting high satisfaction, '2' indicating moderate satisfaction and '3' representing dissatisfaction [8].

The patient was transferred to the operating room (OR), where the medical team diligently monitored their systolic blood pressure (SBP), diastolic blood pressure (DBP), heart rate (HR), oxygen saturation (SpO_2_) and electrocardiogram (ECG) every five minutes throughout the surgery. The patient received a subarachnoid block (SAB) using an injection of bupivacaine heavy 0.5% 12 mg, with aseptic precautions followed while sitting at the L2-L3 or L3-L4 intervertebral space. The patient was instructed to lie down after the procedure, and VASA 1 was recorded. The patient was provided with a scale and pen to mark on a horizontal line as previously explained. The patients now receive noise-cancelling earphones in the operating theatre, which they wear until the completion of the surgery following a successful subarachnoid block. If any patient felt uncomfortable, the music was discontinued, and they were excluded from the study. However, none of the patients were dropped from our study. After the surgical procedure, the earphones were removed, and the patient's VASA 2 and PSS were recorded in the post-anaesthesia recovery room.

Statistical analysis

Based on a previous study, the difference in the mean visual analogue scale for anxiety 2 (μ1-μ2) during a caesarean delivery was 0.49 cm (control group = 1.76 and music group = 1.27), and the population variance (σ) was 1.20. The sample size was calculated as follows: (n) = 2 (Zα/2 + Z [1-β])2 × σ2/(μ1-μ2)2, assuming 0.05 is the level of significance (Zα/2 = 1.96) and 80% power (Z [1-β]) = 0.84) is 94 (Hepp et al., 2018) [9].

Data were analysed with the software Statistical Package for Social Sciences (SPSS) version 23.0 (IBM SPSS Statistics, Armonk, NY). Student's t test was used for quantitative data and the chi-square test for qualitative data. A p-value of ≤0.05 was considered statistically significant.

## Results

**Figure 1:**
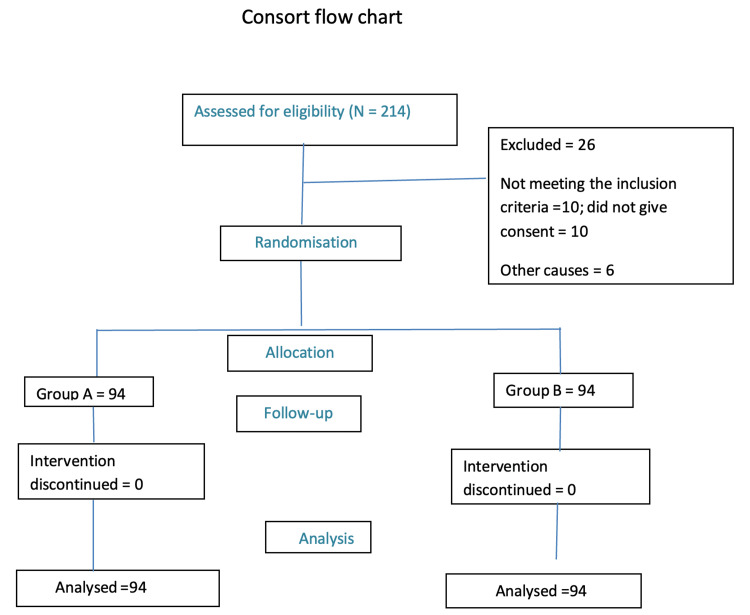
Consort flow chart

A total of 188 patients were included and analysed in our study based on the inclusion and exclusion criteria. None of them were dropped from the study (Figure 1).

In our study, the demographic profile of patients in the two groups was comparable. During the study, no statistically significant difference was observed in HR, SBP and DBP between the two groups (Tables 1-3).

**Table 1:** The comparison of systolic blood pressure among the two groups SD: standard deviation

Serial number	Time	Group A (n = 94)		Group B (n = 94)		Student's 't' test	
		Mean	SD	Mean	SD	't'	'p'
1	Baseline	126.23	12.91	124.15	12.30	0.802	0.425
2	0 minute	123.96	13.52	124.72	13.17	-0.278	0.781
3	5 minutes	116.34	13.55	115.74	13.43	0.214	0.831
4	15 minutes	114.53	12.26	116.13	12.20	-0.633	0.529
5	30 minutes	115.21	13.31	119.70	12.47	-1.687	0.095
6	45 minutes	117.21	11.32	120.81	10.29	-1.612	0.110
7	1 hour	118.15	12.47	122.53	10.08	-1.875	0.064
8	1 hour and 15 minutes	118.79	11.01	122.47	9.77	-1.714	0.090
9	End of surgery	120.13	10.68	123.19	9.11	-1.496	0.138

**Table 2:** The comparison of heart rate among the two groups SD: standard deviation

Serial number	Time	Group A (n = 94)		Group B (n = 94)		Student's 't' test	
		Mean	SD	Mean	SD	't'	'p'
1	Baseline	95.00	19.48	89.68	16.28	1.436	0.154
2	0 minute	95.36	19.47	91.77	16.93	0.955	0.342
3	5 minutes	93.70	18.67	92.36	15.43	0.379	0.705
4	15 minutes	89.81	17.60	91.13	14.24	-0.399	0.691
5	30 minutes	86.32	16.09	89.51	14.25	-1.018	0.311
6	45 minutes	84.89	15.57	89.11	13.71	-1.392	0.167
7	1 hour	84.04	15.31	89.15	13.91	-1.692	0.094
8	1 hour and 15 minutes	83.26	15.04	88.70	13.54	-1.845	0.068
9	End of surgery	83.32	14.01	88.83	13.01	-1.975	0.051

**Table 3:** The comparison of diastolic blood pressure among the two groups SD: standard deviation

Serial number	Time	Group A (n = 94)		Group B (n = 94)		Student's 't' test	
		Mean	SD	Mean	SD	't'	'p'
1	Baseline	76.72	9.49	73.87	12.38	1.253	0.213
2	0 minute	71.91	11.26	73.06	12.43	-0.470	0.640
3	5 minutes	66.62	10.09	67.30	11.76	-0.301	0.764
4	15 minutes	64.38	8.86	66.96	10.34	-1.296	0.198
5	30 minutes	65.13	9.56	68.89	9.13	-1.953	0.054
6	45 minutes	66.64	8.60	69.66	8.94	-1.670	0.098
7	1 hour	68.13	8.98	71.72	8.75	-1.966	0.052
8	1 hour and 15 minutes	68.43	7.87	71.26	8.89	-1.633	0.106
9	End of surgery	69.68	6.85	71.94	8.18	-1.450	0.151

The VASA 1 of both groups was similar, with a mean value of 4.36 ± 1.13 for group A and 4.23 ± 1.05 for group B (p = 0.606). However, group A had lower VASA 2 (1.79 ± 0.83) than group B (3.77 ± 0.98). The difference was statistically significant (p < 0.001) (Table 4).

**Table 4:** Comparison of visual analogue scale for anxiety (VASA) 1 and VASA 2 among the two groups VASA 1: visual analogue scale for anxiety recorded immediately after spinal anaesthesia. VASA 2: visual analogue scale for anxiety recorded in the postoperative period SD: standard deviation

	Group	Number of cases	Minimum	Maximum	Median	Mean	SD
VASA 1	Group A	94	2	8	4	4.36	1.13
	Group B	94	2	6	4	4.23	1.05
	Total	188	2	8	4	4.30	1.08
		Z = 0.515; p = 0.606 (Mann-Whitney U test)					
VASA 2	Group	Number of cases	Minimum	Maximum	Median	Mean	SD
	Group A	94	1	4	2	1.79	0.83
	Group B	94	2	6	4	3.77	0.98
	Total	188	2	6	3	2.78	1.34
		Z = 10.332; p < 0.001 (Mann-Whitney U test)					

The patient satisfaction score in group A was notably higher than those in group B. A total of 52 patients were highly satisfied in group A as compared to none in group B (p < 0.001), and a total of 42 patients were moderately satisfied as compared to eight patients in group B (p < 0.001). Eighty-six patients in group B were unsatisfied compared to none in group A (p < 0.001) (Table 5).

**Table 5:** The comparison of patient satisfaction scores among the two groups

Serial number	Level of satisfaction	Group A (n = 94)		Group B (n = 94)		c^2^; 'p'
		Number	%	Number	%	
1	Highly satisfied	52	55.3	0	0.0	71.882; p < 0.001
2	Moderately satisfied	42	44.7	8	8.5	31.497; p < 0.001
3	Unsatisfied	0	0.0	86	91.5	158.510; p < 0.001

## Discussion

Our study found that patients exposed to music had lower postoperative anxiety levels (VASA 2) compared to those who were not exposed. Additionally, the patients who listened to music reported higher satisfaction scores than the control group. However, the two groups had no significant difference in the haemodynamic parameters.

In line with our research, a randomised controlled trial has shown reduced perioperative anxiety and stress scores among patients who participated in music therapy. The study concluded that music therapy is a safe and effective procedure for reducing anxiety and stress levels in patients [10]. Another study has shown that music therapy can help minimise preoperative anxiety for patients undergoing daycare surgery [11].

Research has shown that listening to music before and after surgery can enhance patient satisfaction and alleviate anxiety and depression [8].

The meta-analysis conducted by Köhler et al. on twenty-one studies clearly demonstrates that music therapy has a significant impact on psychological well-being (p < 0.001), physical symptom distress (p = 0.017) and quality of life (p = 0.023) [12].

Listening to music has been shown to reduce anxiety levels by lowering the levels of various stress hormones and biochemical markers, such as cortisol, epinephrine and norepinephrine. This is due to the neural connections between the auditory pathway and the hypothalamus, hippocampus and reticular activating system. In addition, music provides mental distraction and masks unpleasant noises, contributing to its calming effect.

According to a meta-analysis of 18 studies, perioperative music can help reduce the neuroendocrine cortisol stress response to surgery (p < 0.01) [2].

In a meta-analysis of 47 studies with 2474 participants, the impact of music therapy on physiological and psychological stress-related outcomes was studied. The results showed a considerable medium-to-strong effect (d = 0.723 {0.51-.94}) of music therapy on stress-related outcomes in mental healthcare and medical settings [13].

According to our findings, the patients exposed to music reported a notable increase in their satisfaction scores. This conclusion was also supported by the research conducted by Palmer et al. who divided 207 female patients into three groups. The first group received patient-selected live music before the surgery and therapist-selected recorded music during the surgery. The second group received patient-selected recorded music before the surgery and therapist-selected recorded music during the surgery. The third group received the usual care before the surgery and wore noise-blocking earmuffs during the surgery. Both the music groups showed significant reductions in anxiety scores and improvement in patient satisfaction scores compared to the control group during the surgery [14].

Music therapy has been used since 1940 to enhance relaxation and well-being among chronically ill patients and children [15]. Using pre-recorded music for therapeutic purposes is commonly known as music medicine. This approach involves patients participating in passive listening sessions that medical professionals provide [16].

Listening to music can help with recovery and reduce anxiety, depression and pain by triggering relaxation signals in the brain. Different genres of music can also affect cortisol levels and boost immune system components such as IgA and natural killer cells [17-19].

Listening to slow and repetitive music can help reduce anxiety by inducing relaxation and altering the consciousness. This physiological response is incompatible with stress, leading to decreased perceived anxiety levels and more relaxed physiological reactions. Music occupies attention channels in the brain, ultimately reducing anxiety by diverting attention from stressful environmental stimuli [20].

Our study had limitations, including a single-centric design, a smaller sample size, only one type of study and the consideration of only one gender. We recommend conducting more extensive multicentric trials to reach a more definitive conclusion.

## Conclusions

It can be concluded that playing pre-approved music at an appropriate volume can reduce anxiety levels and increase satisfaction ratings for patients undergoing abdominal hysterectomy surgeries. This is likely due to the music's ability to boost the immune response, distract from stressful environmental stimuli and decrease stress hormone levels during the procedure.
